# Evaluating the Biodeterioration Enzymatic Activities of Fungal Contamination Isolated from Some Ancient Yemeni Mummies Preserved in the National Museum

**DOI:** 10.1155/2014/481508

**Published:** 2014-11-13

**Authors:** Khalid Mohammed Naji, Qais Yusuf M. Abdullah, Aida Qaseem M. AL-Zaqri, Saeed M. Alghalibi

**Affiliations:** ^1^Department of Chemistry, Faculty of Science, Sana'a University, Sana'a, Yemen; ^2^Department of Biology, Faculty of Science, Sana'a University, Sana'a, Yemen; ^3^Department of Archaeology, Faculty of Art, Sana'a University, Sana'a, Yemen

## Abstract

Sophisticated mummification using chemical preservation was prevalent in ancient Yemeni civilization as noted in the 4th century B.C. mummies of the National Museum of Yemen, Sana'a, used in this study. Five of these mummies were used to evaluate hydrolytic enzymes produced as a result of fungal contamination. Forty-seven fungal species were isolated, thereby reflecting a high degree of contamination which may have resulted from the poor ventilation and preservation system. *Aspergillus* was the most common genus isolated (48.9%). Fifteen isolates exhibited ability to produce cellulase (EC; 3.2.1.4), *Aspergillus candidus* being the highest cellulose-producer. Pectin lyase (PL, EC; 4.2.2.2) and pectin methyl esterase (PME, EC; 3.1.1.11) were produced by *Trichoderma hamatum*, whereas chitinase (EC; 3.2.1.14) was produced by *Aspergillus niger*. Protease activity was noted by only *Cladosporium herbarum*. The higher activities of these fungal hydrolytic enzymes represent the major threats of biodeterioration including deteriorating linen bandages as well as the mummy bodies. Therefore, it is recommended to improve the preservation system of the mummies at the National Museum to minimize the contamination up to the lowest level and protect the mummies from biodeterioration.

## 1. Introduction

Human mummies are extremely susceptible to damage from environmental factors [1]. There are a number of abiotic and biotic factors, such as pollution, light, humidity, temperature, microorganisms, and insects, that have deteriorating effects on museum materials [2]. The most serious damage on mummies occurs by microorganisms such as bacteria, actinomycetes, and fungi [1, 3, 4].

In an indoor environment, fungi are the most hazardous microorganisms responsible for the biocontamination of museum collections. Unlike bacteria whose growth in museums is limited by their need for water, fungi are able to grow at relatively low levels of temperature and humidity such as those usually maintained in the museums [5, 6]. Even in quite harsh conditions including dryness and low temperature, fungal spores are able to survive for a long time [7]. Fungi are usually present in display and storage conditions of museums [8, 9].

Fungal degradation is one of the highest risk factors for deterioration of ancient mummies [10, 11]. The deterioration by fungi seems to be a predominant feature in the museums of many countries all over the world [8, 12]. Fungi excrete enzymes that digest organic matter, thereby altering and weakening those materials. In addition, many molds contain colored substances that can cause stains and spots on textiles and decrease the strength of the fabric. Many fungi can be dangerous to people and in some cases pose a major health hazard and cause zoonotic superficial infections as a consequence of invading keratinized tissues of skin, hair, and nails [13, 14].

Fungal biodeterioration results in a change in the quality or value of a material and makes it less functional in utilization terms. A battery of extracellular hydrolytic enzymes excreted by fungi results in the formation of acidic products that cause chemical alteration of the material under attack [15]. Cellulases catalyze hydrolysis of cellulose, break internal bonds to disrupt its crystalline structure, and expose individual cellulose polysaccharide chains [16]. Pectinases catalyze hydrolysis of pectic substances readily soluble in water. Pectolytic enzymes are divided into two classes, namely, pectinesterases and pectin depolymerases. The former enzyme deesterifies pectin by the removal of methoxy residues and is called pectin methoxy esterase (PME), while the latter splits the main chain and is further classified as polygalacturonase (PG) and pectin lyases (PL) [17–19]. Chitinases are glycosyl hydrolases having the ability to degrade *β*-1, 4-linked insoluble linear polymers of* N*-acetylglucosamine (chitin) directly to low molecular weight chitooligomers [20]. Proteases, a complex group of hydrolytic enzymes, hydrolyze proteins into small peptides and amino acids by the addition of water across amide bonds. They have evolved different classes of proteases that perform the same reaction by using completely different catalytic mechanisms. Fungi are known to produce acid, neutral, alkaline, and metalloproteases. A single organism can produce more than one type of protease [21]. Fungal proteases are active over a wide pH range (pH 4 to 11) and exhibit broad substrate specificity [22].

The ancient Yemeni mummies discovered in 1983 in mountain caves near* Shebam al-Giras *were rescued by the Archeology Department Expedition of University of Sana'a [23]. The Yemeni mummies are different from those of Egypt. They are older and go back to the 4th century B.C. according to results of radiocarbon analysis of the leather and linen wrappings [24]. The dead bodies were treated different from the Egyptian ones. Local materials used in mummification to absorb moisture such as raá plant (*Aerva javanica*), henna (*Lawsonia inermis*), myrrh, and other aromatic plants were used to preserve the corpses. Oxides of iron and sulfur were used to fix proteins in the corpse. The process was completed with the corpses being wrapped in linen (*Linum usitatissimum*) bandages. The bandaging took place over an elongated period of days [25]. Most of the materials used in the mummification are natural organic substances. Hence, these mummies are liable to deterioration. In addition, the storage conditions of the mummified corpses in the museum display are in unsuitable environmental conditions. These mummies are of special importance as they depict ancient Yemeni civilization and its use of advanced medical and scientific knowledge in the preservation of the corpses.

This study aimed at screening of fungal contamination of five Ancient Yemeni mummies preserved in the National Museum of Yemen at Sana'a. The fungal hydrolytic enzymes assessed were cellulase, PL, PME, chitinase, and protease. This in turn will help in our understanding for the use of better preservative conservation procedures in order to safeguard the mummies from future damage.

## 2. Materials and Methods

### 2.1. Mummy Samples

Five mummies preserved in the National Museum of Yemen at Sana'a were used in this study for the collection of fungal contamination. The details of the mummies used are as described in Table 1.

### 2.2. Screening of Fungal Contamination

Samples were taken from different skin areas (face, hands, and feet) of the five mummies. Fungi were isolated using the conventional methods of swabbing and streaking [26] on Potato Dextrose Agar (PDA). Plates incubated at 27 ± 1°C for 7 days. Three replicates were prepared for each sample. The resultant colonies were isolated, purified, and identified. Pure cultures of the identified fungi were transferred to a slant and used for physiological studies. Fungi were identified based on their morphology according to Raper and Fennell [27], Ellis [28], and Barnett and Hunter [29].

### 2.3. Screening the Hydrolytic Enzymatic Activity of Isolated Fungi

Twenty-one isolates were examined for their ability to produce common hydrolytic enzymes. Standard methods were used for assay of the following enzyme activities: cellulase [30], pectinase [31], chitinase [32], and protease [33].

### 2.4. Production of Enzymes in Liquid Nutrient

The isolates that were found to exhibit highest activity of each enzyme on solid media were grown in liquid media for further analysis.

### 2.5. Growth Condition

The fungi were grown in Erlenmeyer flasks (100 mL) containing 25 mL of the liquid growth medium. After sterilization, the flasks were inoculated with a single 6 mm disc cut out from the margin of 5 d colony of the fungus grown on Czapek's agar medium. The inoculated flasks were incubated as stationary culture at 30°C for 7, 14, and 21 days [34]. The experiments were carried out in triplicate.

### 2.6. Growth Medium

*Cellulase*. 100 mL of the liquid medium contained cellulose, 1 g; (NH_4_)_2_SO_4_, 0.14 g; K_2_HPO_4_, 0.6 g; KH_2_PO_4_, 0.20 g; and MgSO_4_·7H_2_O, 0.01 g in distilled water [35]. An isolate of* Aspergillus candidus* was inoculated into sterile media for the production of cellulase.

*Pectinase*.* Trichoderma hamatum* isolate was used for the production of PL and PG enzymes using the medium described by Eggins and Pugh [36]. The composition per 1000 mL was pectin, 10 g; L-asparagine, 0.5 g; (NH_4_)_2_SO_4_, 0.5 g; KH_2_PO_4_, 1.0 g; KCl, 0.5 g; CaCl_2_, 0.2 g; yeast extract, 0.5 g; MgSO_4_·7H_2_O, 0.2 g in distilled water. The pH was adjusted to 5 using acetate buffer.

*Chitinase*.* Aspergillus niger *isolate was used for the production of chitinase using the medium described by Hsu and Lockwood [32]. 100 mL of liquid growth medium contained NaNO_3_, 0.5 g; KH_2_PO_4_, 0.2 g; KCl, 0.1 g; yeast extract, 0.05 g; MgSO_4_ 7H_2_O, 0.05 g in distilled water. The pH was adjusted to 5 using acetate buffer.

*Protease*.* Cladosporium herbarum *isolate was used for protease production in liquid medium as described by Ali [37]. 1000 mL of liquid growth medium contained K_2_HPO_4_, 1 g; MgSO_4_·7H_2_O, 0.5 g; KCl, 0.5 g; FeSO_4_·7H_2_O, 0.01 g; sucrose, 30 g; casein, 1% (w/v) in distilled water. The pH was adjusted to 5 using acetate buffer.

### 2.7. Enzymes Assay

The culture filtrates were used as crude enzyme preparations for all mentioned enzymes.

*Cellulase* activity was measured by estimating the liberated amount of reducing sugar using dinitro salicylic acid (DNS), according to Miller [38] with the modification of Bernfeld [39] using glucose for the standard curve. The absorbance was read at 540 nm using boiled enzyme as control. One unit of cellulase activity is defined as the quantity of enzyme that catalyzes the liberation of 1 *μ*mol/min of reducing sugar measured using glucose as standard.

*Pectinase*. Two enzymes of pectinase family were estimated.

- *Pectin lyase (PL)* activity was assayed spectrophotometrically by determining uronide at 235 nm [40]. 3 mL of reaction mixture contained 0.1% pectin in 0.05 M of Tris-HCl buffer (pH 8) and 0.5 mL of culture filtrate. The reaction mixture was incubated at 30°C for 3 hours. Then 3 mL of 0.01 N HCl and 1 mL of reaction mixture were mixed and the optical density read. One unit of PL activity is defined as the amount of enzyme which causes an increase in absorbance of 0.01 in 30 min.
- *Pectin methyl esterase (PME)* activity was assayed based on the method described by Kertesz [41] and modified by Olutiola and Akintunde [42]. In sterilized 25 mL conical flask, 2 mL of culture filtrate was mixed with 5 mL of 1.2% pectin (in 0.05 M Tris-HCl buffer containing 5 mM sodium carbonate, pH 8). The flask was incubated in water bath at 30°C for 3 h followed by titration with 0.02 N NaOH to adjust pH to 8. One unit of PME activity was defined as the amount of enzyme required for addition of 1 *μ* equivalent of NaOH per hour to maintain the reaction at pH 8.

*Chitinase* activity was estimated according to Monreal and Reese [43]. 5 mL reaction mixture contained 1% colloidal chitin in phosphate buffer (pH 5) and 1 mL of culture filtrate. The mixture was incubated at 37°C for 1 h, reaction arrested by adding 0.5 mL of DNS, boiled for 15 min in a water bath, and cooled to room temperature. Optical density was then measured at 540 nm against a suitable blank. The reducing sugar released was determined from the standard curve of glucose. One unit of chitinase activity is defined as the amount of enzyme, which produced 1 *μ*mol of reducing sugar min^−1^ under assay conditions.

*Protease* activity was estimated by the method of Folin and Ciocalteu [44]. The reaction mixture containing 5 mL of 0.65% casein (pH 7.5) and 1 mL of enzyme extract was mixed well by swirling and incubated at 37°C for 10 min. The reaction was arrested by adding 5 mL of cold 10% trichloroacetic acid and incubated at 37°C for 30 min. It was filtered through Whatman filter paper and the filtrate thus obtained was used for color development. 5 mL of Na_2_CO_3_ and 1 mL of Folin-Ciocalteu's (FC) reagent were added to an aliquot of the filtrate and incubated at 37°C for 30 min and absorbance was read at 660 nm. The amount of amino acids released was calculated using standard curve of tyrosine. One unit of protease activity is defined as the amount of enzyme that produced 1 *μ*mol of tyrosine min^−1^ under assay conditions.

*Protein Content*. Soluble protein was estimated according to Lowry et al. method [45], using bovine serum albumin (BSA) as standard.

### 2.8. Determination of Dry Weight

Fungal mycelia were separated from the growth medium by filtration through Whatman no. 1 filter paper. The mycelia were then washed and dried in an oven at 60°C for 24 h. The dry weight of the mycelia was recorded.

### 2.9. Statistical Analysis

Two-way analysis of variance (ANOVA) was used for statistical evaluation of enzymes activities and dry weight by using GraphPad Prism version 6. Data were shown as mean ± SD. The value of *P* < 0.05 was considered statistically significant.

## 3. Results and Discussion

**Figure 1:**
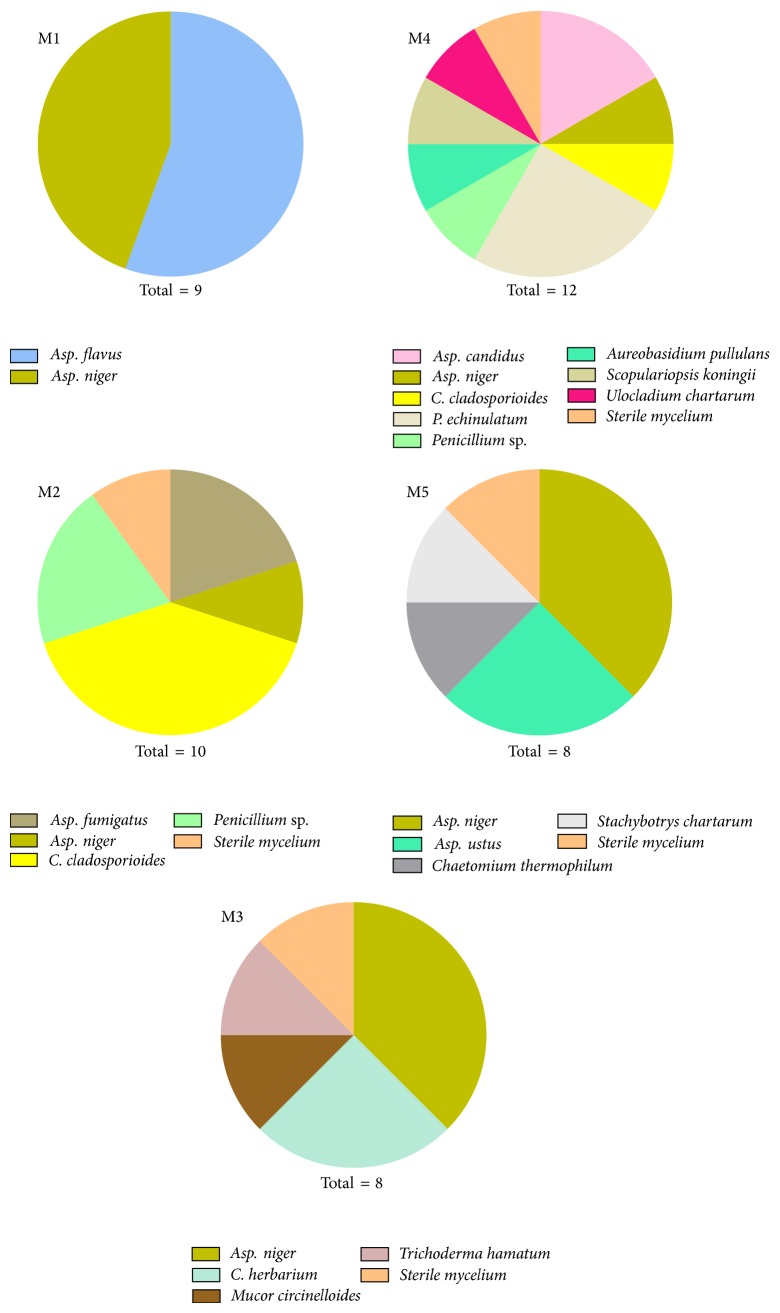
Percentage of mummy's contamination by different species of fungi (M1 = mummy sample no. 1; M2 = mummy sample no. 2; M3 = mummy sample no. 3; M4 = mummy sample no. 4; M5 = mummy sample no. 5; Total indicates the total no. of fungal species isolated from each mummy).

Fifteen species belonging to 10 genera of fungi were isolated from five ancient Yemeni mummies on PDA. The total count of fungi isolated from all samples was 47 (Table 2). Mummy 1 (M1) had the lowest fungal contamination, that is, only two species of* Aspergillus* (Figure 1). In contrast, M4 showed the highest contamination with 12 isolates of 9 species (Figure 1). The other mummies showed moderate contamination. According to the numbers of isolated fungi from the five mummies, the order from higher to lower contamination is as follows: M4 > M2 = M5 > M3 > M1 (Figure 1).

The diversity of fungal profile of each mummy, although preserved in the same museum, may have resulted from environmental conditions, which differ in each room inside the museum due to several factors including the construction and design of the building. Also, the place from which the mummies were unearthed varies. Each mummy was preserved in a separate room, and preservation conditions vary from room to room in terms of ventilation, humidity, and temperature (data not shown). In addition to that, some mummies were treated by disinfectant whereas others were not.

*Aspergillus* was the most common genera isolated from the mummy samples with high frequency of 48.94% of the total fungal count, the most common species being* A. niger* (25.53%) followed by* A. flavus* (10.63%), while* A. fumigatus, A. candidus,* and* A. ustus *were isolated in low frequency.* Cladosporium *and* Penicillium *were isolated in moderate frequency representing 14.89% and 12.76%, respectively.* Aureobasidium pullulans, Chaetomium thermophilum, Mucor circinelloides, Scopulariopsis koningii*,* Stachybotrys chartarum*,* Trichoderma hamatum,* and* Ulocladium chartarum* were isolated in rare incidence from mummy samples.

Our results exhibited similar fungal contamination when compared to isolates from Egyptian mummies. These included* Alternaria tenuis*,* Aspergillus humicola*,* Aspergillus niger*,* Chaetomella horrid*,* Chaetomium globosum*,* Hormodendrum viride*, and* Penicillium corylophilum* [11]. Twenty-three mummies from cemeteries and caves of northern Mexico were found to have mainly* Penicillium*,* Aspergillus*,* Cladosporium*,* Alternaria*,* Candida tropicalis,* and* Candida albicans* [46]. Also three species of* Aspergillus* and one of* Alternaria* were found to be associated with open deteriogens skeleton of giraffe in zoological museum of Punjab University in Lahore India [47].

**Figure 2:**
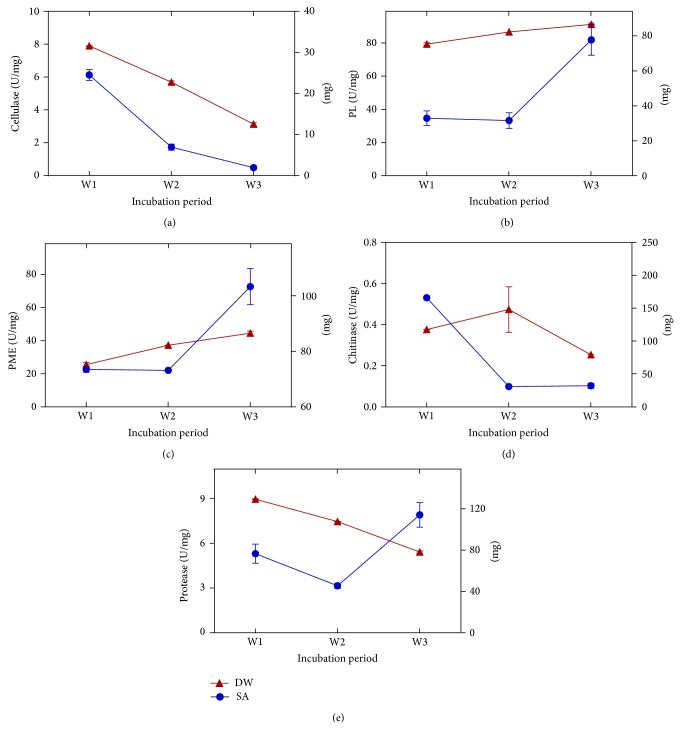
Levels of specific activity (SA) (U/mg protein) during growth incubation at 28°C (left *Y*-axis) for (a) cellulase from* Aspergillus candidus,* (b) chitinase from* Aspergillus niger,* (c) protease from* Cladosporium herbarum*, (d) pectin lyase (PL) from* Trichoderma hamatum,* (e) pectin methyl esterase (PME) from* Trichoderma hamatum.* Right *Y*-axis indicates the effect of incubation period on dry weight (DW) of each fungus growth. Data presented in these graphs are mean ± standard division for double measures of three separate repeats.

Twenty-four isolates were examined for their ability to produce cellulase, pectinase, chitinase, and protease. Among these, 15 isolates exhibited enzymatic potential (Table 3). Cellulase enzymes degrade cellulose to disaccharides, while endoglucanases cut the cellulose chain in a random fashion and exoglucanases successively remove single cellobiose or glucose units from the nonreducing end of the cellulose chain [10]. In this study* Aspergillus candidus, Aureobasidium pullulans, Ulocladium chartarum, Cladosporium cladosporioides, A. ustus, *and* Penicillium *sp., respectively, exhibited high ability to produce cellulolytic activity based on the zone area. Similar results showed that* Penicillium *sp. produced high activity of cellulose [48]. On the other hand, enzyme extract obtained from* A. niger* and* A. terreus* was found to be rich in *β*-glucosidase [49].* Aspergillus candidus *was prepared for quantitative estimation of cellulase; it showed significant reduction in cellulase activity with time of incubation period (Figure 2(a)). Ancient Yemeni mummies are ~2000 years old, and their mummification procedure contains many botanical materials. Plenty of species able to produce cellulase suggest that this enzyme is the main cause of degradation of linen bandages of mummies and other materials.

Pectinolytic activity is one of the most important extracellular enzymes of fungi which hydrolyzes pectic substance commonly present in plant cell walls [50]. Our results revealed that, among the tested fungi,* Penicillium echinulatum and Trichoderma hamatum *showed the highest pectolytic activity for both pectinases enzymes, pectin methyl esterase (PME) and pectin lyase (PL). These results are in agreement with many studies that have shown* Trichoderma* sp. [51] and* Pencillium* sp. to be the most common pectinase producers [50]. In contrast,* Aspergillus *sp. showed the highest pectinolytic activity [52] followed by* Scopulariopsis koningii *and* Mucor circinelloides*. Activities of PL and PME from* Trichoderma *hamatum did not show any difference in the specific activity of both enzymes in the second week of incubation (Figures 2(b) and 2(c)), but in the third week both showed significant elevation (Table 4). This suggests that pectinase enzymes are involved in fungi metabolism and may help in its growth adaption. These enzymes play an important role in deteriorating linen bandages as well as the mummy.

Results of our study show that* A. niger* and* P. citrinum *were able to produce chitinase enzyme. These results are similar to high chitinolytic activity of* A. niger* [53]. However,* Trichoderma viride* showed the maximum chitinolytic activity from eight fungal species belonging to 5 genera isolated from agricultural soil sample [54]. The activity of chitinase produced by* A. niger* showed acute reduction in the second week of incubation time when compared to the first week (Figure 2(b)). This suggests that chitinase enzyme of* A. niger* isolated from mummy skin is not involved in the growth adaptation of the isolates.

Proteolytic enzymes are a large group of enzymes that cleave the peptide bonds of proteins to small fragments and amino acids. Fungi cause proteolysis of collagen which is dependent on many factors such as storage and environmental conditions, in addition to presence of certain substances that reside on the mummy's skin [10]. In our results, only one fungal isolate was able to produce proteolytic activity (Table 2). This isolate was* C. herbarium*. The proteolytic activity was found to reduce after 2 weeks of incubation whereas, after 3 weeks, it was elevated by 2-fold (Figure 2(c)). In contrast, the fungi from different source than mummy showed higher proteolytic activity. 72.2% of 54 fungal isolates showed proteolytic activity [55].

Identifying the fungal species and the factors enhancing their growth is an essential step for the success in establishing a proper and effective strategy for preservation of the collection of mummies in museums. The fungal problem should be addressed either by controlling physical conditions of the surrounding environment in which they are stored/displayed or by the treatment with biocides. Control of the environmental conditions is the best means of protecting mummies.

## 4. Conclusion

It can be concluded that the higher degree of contamination of the ancient Yemeni mummies preserved in the National Museum of Yemen may result from the poor level of preservation conditions. Also the higher activity of hydrolytic enzymes including cellulase, PL, PME, chitinase, and protease from the isolated fungi species represents the major threat that can cause biodeterioration of the ancient Yemeni mummies. Therefore, it is recommended to improve the preservation systems of the mummies at the National Museum to minimize the contamination to the lowest level, which in turn will protect the mummies from biodeterioration.

## Figures and Tables

**Table 1:** List of ancient mummy's samples collected from National Museum of Yemen at Sana'a (NMS) during this investigation.

No.	Mummy description	Source	Age years
M1	A male skeleton of mummy dried naturally (dehydrated body)^*^	*Shebam al-Giras*-Sana'a	~2000

M2	Human mummified remains dried naturally (dehydrated body) showing mold deterioration	Al-Hymah-Sana'a	~2000

M3	A typical Yemeni mummy wrapped with linen cloth	Al-Tawilah Al-Mahwit	~2000

M4	Severe fragile and damaged human mummified remains	Al-Tawilah Al-Mahwit	~2000

M5	Human mummified body dried naturally (dehydrated body) showing linen textiles without a conservation process. Deterioration of textiles eventually causes complete loss	Al-Tawilah Al-Mahwit	~2000

^*^A mummy embalmed or treated with chemicals, or it may have been naturally desiccated under extreme cold, dryness, or even lack of air.

**Table 2:** Fungal species isolated from some ancient Yemeni mummies collection of National Museum in Sana'a.

Fungi isolated	M1	M2	M3	M4	M5	Sp. T.C	T.C	%
***Aspergillus* sp.**							23	**48.94**
*A. candidus *	—	—	—	2	—	2		4.26
*A. flavus *	5	—	—	—	—	5		10.64
*A. fumigatus *	—	2	—	—	—	2		4.26
*A. niger *	4	1	3	1	3	12		25.53
*A. ustus *	—	—	—	—	2	2		4.26
***Cladosporium* sp.**							7	**14.89**
*C. herbarium *	—	—	2	—	—	2		4.26
*C. cladosporioides *	—	4	—	1	—	5		10.64
***Penicillium* sp.**							6	**12.76**
*P. echinulatum *	—	—	—	3	—	3		12.76
*Penicillium *sp.	—	2	—	1	—	3		12.76
***Aureobasidium pullulans***	—	—	—	1	—		1	**2.13**
***Chaetomium thermophilum***	—	—	—	—	1		1	**2.13**
***Mucor circinelloides***	—	—	1	—	—		1	**2.13**
***Scopulariopsis koningii***	—	—	—	1	—		1	**2.13**
***Stachybotrys chartarum***	—	—	—	—	1		1	**2.13**
***Trichoderma hamatum***	—	—	1	—	—		1	**2.13**
***Ulocladium chartarum***	—	—	—	1	—		1	**2.13**
**Sterile mycelium **	—	1	1	1	1		4	**8.5**

Total	9	10	8	12	8		47	

M = mummy; NI = number of isolates; T.C = total count; % = percentage of occurrence.

**Table 3:** Screening of enzymatic activity of some fungal species isolated from ancient Yemeni mummies.

Fungal species	NIT	Cellulase	Pectinase		Chitinase	Protease
			PL	PME		
*Aspergillus candidus *	2	H	N.D	H	N.D	N.D
*Aspergillus flavus *	1	N.D	N.D	N.D	N.D	N.D
*Aspergillus fumigatus *	1	N.D	N.D	N.D	N.D	N.D
*Aspergillus niger *	6	N.D	N.D	N.D	M	N.D
*Aspergillus ustus *	1	H	N.D	N.D	N.D	N.D
*Cladosporium herbarium *	1	N.D	N.D	N.D	N.D	M
*Chaetomium cladosporioides *	1	H	N.D	N.D	N.D	N.D
*Pencillium echinulatum *	1	N.D	H	H	N.D	N.D
*Pencillium *sp.	1	N.D	N.D	N.D	M	N.D
	2	H	N.D	N.D	N.D	N.D
*Aureobasidium pullulans *	1	H	N.D	N.D	N.D	N.D
*Chaetomium thermophilum *	1	M	N.D	N.D	N.D	N.D
*Mucor circinelloides *	1	W	M	H	N.D	N.D
*Scopulariopsis koningii *	1	N.D	H	N.D	N.D	N.D
*Stachybotrys chartarum *	1	M	M	N.D	N.D	N.D
*Trichoderma hamatum *	1	N.D	H	H	N.D	N.D
*Ulocladium chartarum *	1	H	N.D	N.D	N.D	N.D

NIT = number of isolates tested; PL = pectin lyase; PME = pectin methyl esterase; N.D = no enzyme was detected; W = weak >0.5; M = moderate 0.5–0.9; H = high <10 mm.

**Table 4:** Two-way ANOVA statistical test of comparative enzymes specific activities (SA) during incubation period.

	*P* value		
	W2 versus W1	W3 versus W1	W3 versus W2
Cellulase	<0.0001	<0.0001	0.0047
PL	0.9533	0.0013	0.0011
PME	0.9905	0.0011	0.0010
Chitinase	<0.0001	<0.0001	0.8624
Protease	0.0247	0.0129	0.0014

W1 = after one week; W2 = after 2 weeks; W3 = after 3 weeks.
